# Ultraviolet irradiation increases size of the first clutch but decreases longevity in a marine copepod

**DOI:** 10.1002/ece3.5510

**Published:** 2019-08-01

**Authors:** Kyle B. Heine, Matthew J. Powers, Christine Kallenberg, Victoria L. Tucker, Wendy R. Hood

**Affiliations:** ^1^ Department of Biological Sciences Auburn University Auburn AL USA

**Keywords:** hormesis, life history, *reproductive performance*, stress, Tigriopus californicus

## Abstract

An important component of life history theory is understanding how natural variation arises in populations. Both endogenous and exogenous factors contribute to organism survival and reproduction, and therefore, it is important to understand how such factors are both beneficial and detrimental to population dynamics. One ecologically relevant factor that influences the life history of aquatic organisms is ultraviolet (UV) radiation. While the majority of research has focused on the potentially detrimental effects that UV radiation has on aquatic organisms, few studies have evaluated hormetic responses stimulated by radiation under select conditions. The goal of this study was to evaluate the impact of UV‐A/B irradiation on life history characteristics in *Tigriopus californicus* copepods. After exposing copepods to UV‐A/B irradiation (control, 1‐, and 3‐hr UV treatments at 0.5 W/m^2^), we measured the impact of exposure on fecundity, reproductive effort, and longevity. We found that UV irradiation increased the size of the first clutch among all reproducing females in both the 1‐ and 3‐hr experimental groups and decreased longevity among all females that mated in the 1‐hr treatment. UV irradiation had no effect on the number of clutches females produced. These findings indicate a potential benefit of UV irradiation on reproductive performance early in life, although the same exposure came at a cost to longevity.

## 1 INTRODUCTION

The life history of sexually reproducing organisms can vary greatly both within and between populations and is influenced by numerous endogenous and exogenous factors (Fisher, 1930; Stearns, 2000). To understand this variation, it is important to delineate how the environment influences survival and reproductive success. For organisms to survive and achieve reproductive success in the face of changing environments, they must balance the costs and benefits of exogenous stressors with maintaining basic metabolic functions, surviving to reproductive age, and producing viable offspring (Speakman, 2008; Williams, 1966). Many exogenous stressors to which organisms are exposed can have beneficial effects on organism performance at certain levels of exposure but detrimental effects at other levels of exposure. This dichotomy can make it challenging for an investigator to predict the impact of a stressor on animal performance.

A hormetic response improves organismal performance under low levels of exposure to a stressor that is detrimental at higher levels of exposure (Hood, Zhang, Mowry, Hyatt, & Kavazis, 2018; Mattson, 2008; Yun & Finkel, 2014). Hormetic responses are ubiquitous (Constantini, 2014), occurring in varied organisms, including insects (Shephard et al., 2018), humans (Radak, Chung, Koltai, Taylor, & Goto, 2008), and rodents (Zhang, Humes, Almond, Kavazis, & Hood, 2017), and they have been shown to occur as a consequence of both endogenous and environmental stressors (Zhang & Hood, 2016). One such factor that is often considered harmful but can also benefit organism performance at low levels of exposure is ultraviolet (UV) radiation (Hessen, 2008; Hylander, Grenvald, & Kiørboe, 2014; Paul & Gwynn‐Jones, 2003; Williamson, Neale, Grad, De Lange, & Hargreaves, 2001). In mammals, the interaction between UV‐B radiation and intracellular catalase produces reactive oxygen species (ROS; Heck, Vetrano, Mariano, & Laskin, 2003). ROS are highly reactive molecules that can directly damage DNA, lipids, and proteins. High levels of UV irradiation can directly damage DNA (Boyle & Setlow, 1970; Cadet, Sage, & Douki, 2005; Setlow & Setlow, 1962) and can indirectly reduce cellular performance via ROS‐induced cellular damage (Finkel & Holbrook, 2000). However, lower levels of ROS have been shown to serve as a cellular signal to increase antioxidants, repair enzymes, and stimulate mitochondrial biogenesis (Zhang et al., 2017). This hormetic response can increase cellular performance. Whether UV radiation can have a hormetic effect on organism performance, and therefore, a beneficial effect on the life history of organisms, remains to be further explored.

Ultraviolet radiation is a prime example of an exogenous factor that is ecologically relevant for small‐bodied invertebrates. Copepods found near the water's surface are naturally exposed to UV radiation. While UV radiation has been shown to negatively affect copepod reproduction and survival (Kane & Pomory, 2001; Puthumana et al., 2017; Scott, 1995; Won et al., 2014, 2015), most studies have evaluated the effect of UV radiation at relatively high levels of exposure. Following Han, Puthumana, Lee, Kim, and Lee (2016), who demonstrated increased antioxidant production in *Tigriopus* copepods following 3‐hr UV irradiation at 0.5 W/m^2^, we hypothesized that UV irradiation increases organism performance through enhanced life history characteristics.

We evaluated the impact of UV irradiation on reproductive performance and longevity in the temperate splash zone copepod *Tigriopus californicus* using various performance correlates. Copepods were exposed to 0‐, 1‐, or 3‐hr UV‐A/B irradiation at an intensity of 0.5 W/m^2^. We quantified the number of offspring (nauplii) produced in the first clutch for each female to estimate fecundity (Barreto & Burton, 2013). Given that clutch size and gestation duration (i.e., incubation) often co‐vary (Brown & Shine, 2009; Dobbs, Styrsky, & Thompson, 2006; Okkens et al., 2001), we also quantified the gestation duration of the first clutch. We evaluated the impact of UV irradiation on reproductive effort by quantifying the total number of clutches produced per female. Finally, we measured the impact of UV irradiation on longevity. Assuming a beneficial response, we predicted that fecundity, reproductive effort, and longevity would increase under UV irradiation.

## 2 MATERIALS AND METHODS

### 2.1 Copepod husbandry

This study took place from September 2017 to August 2018. *T. californicus* copepods were obtained from Reef Nutrition, Campbell, CA in two phases—September 2017 and January 2018. Panmictic cultures of *T. californicus* were maintained in 739 ml containers with artificial sea water (ASW) of salinity S = 32 and fed *Isochrysis* and *Tetraselmis* algae ad libitum. Individuals were kept on a natural, ambient light cycle from laboratory windows at 20–23°C. Herein, we refer to these as stock cultures.

### 2.2 Data collection

Male *T. californicus* clasp and guard virgin females until they become reproductively mature (Burton, 1985). Mating clasped pairs were collected from stock cultures and placed into a 24‐well plate half‐filled with ASW of salinity S = 32. For irradiation, the plate was placed inside a black bin with a UV light (wavelengths ≥290 nm; Exo Terra 10.0 UVB Repti Glo Desert Terrarium Lamp) overhead and covered with a black drape to remove effects of ambient light. The majority of photons from the UV light were derived from UV‐A radiation between 340 and 370 nm, decreasing in exposure up to 400 nm and down to UV‐B radiation at 290 nm. Lamp distance from the plate was predetermined to produce an intensity of 0.5 W/m^2^ (Han et al., 2016), measured using a Sper Scientific UV‐A/B light meter. Clasped pairs were randomly assigned to short (1‐hr) and long‐term (3‐hr) UV treatments (Han et al., 2016), or a 1‐hr, full‐spectrum control treatment (no UV‐B produced, placed at a distance so that no UV‐A was detected; Exo Terra Full Spectrum Natural Daylight Bulb). All females were irradiated while clasped by males to ensure that females had not previously mated, allowing us to manipulate which females would or would not mate following treatment. After irradiation, clasped pairs were placed into 100 × 15 mm petri dishes with ad libitum algae and exposed to indirect, natural light from laboratory windows; UV radiation was measured at 0.0 W/m^2^ at this location in the laboratory on a clear, sunny day. Therefore, copepods were only exposed to UV radiation during the aforementioned treatments. All petri dishes were aerated by hand each day. Water salinity was checked weekly, and fresh ASW was added to dishes each week to replace any water loss due to evaporation. Males were removed from petri dishes once females became gravid, and all nauplii were placed back into stock culture once counted. To prevent insemination within a subset of females, virgin females were separated from males during mate guarding immediately after treatment. This allowed us to determine the effects of UV irradiation on longevity between mating and nonmating (virgin) females.

To examine effects of UV irradiation on fecundity, we recorded the number of nauplii produced from the first clutch and the number of days from the appearance of an egg sac to hatching (i.e., gestation) of the first clutch. We assessed reproductive effort and survival by measuring the number of clutches (egg sacs) produced and longevity. *T. californicus* females mate while undergoing five copepodid molts (Burton, 1985; Raisuddin, Kwok, Leung, Schlenk, & Lee, 2007). To control for age at the time of irradiation, the number of molted exoskeletons in each petri dish was quantified once females became gravid. Molts remained in petri dishes throughout the experiments and were checked again once females were deceased. That value was subtracted from five and included as a covariate in longevity models.

### 2.3 Analytical design

All analyses were performed using R version 3.5.0 (R Core Team, 2018). We used the “MASS” library (Venables & Ripley, 2002) for modeling and the “ggplot2” package (Wickham, 2009) for graphical development.

The number of clutches per female was modeled as a dependent variable relative to treatment (control, short, and long UV irradiation) as an independent variable using zero‐inflated negative binomial regression due to over‐dispersed, discrete count data containing excess zeros—likely due to UV‐induced sterility and/or unsuccessful mating. The number of nauplii produced in the first clutch was modeled as a dependent variable relative to treatment using a Poisson generalized linear model (GLM) due to skewed count data containing zeros (Hu, Pavlicova, & Nunes, 2011). The fully saturated model included an interaction between treatment and gestation duration as independent variables and was reduced using the “step” function. The final model was compared to both the null model and the fully saturated model using χ^2^ analysis.

Nonzero counts of gestation duration and longevity were log‐transformed and square‐root transformed, respectively, to achieve residual normality. Gestation duration and longevity were modeled as dependent variables using general linear models (LMs) with treatment as an independent variable. To evaluate the effect of UV irradiation on the trade‐off between reproduction and longevity, we modeled the interactive effect of UV irradiation and reproduction on longevity by comparing female copepods that reproduced, those that did not reproduce, and virgins that did not mate—longevity was not transformed in this model.

We also modeled longevity as a dependent variable with the interaction between the number of clutches and treatment while controlling for age—in this model, longevity was square‐root transformed and included all females that mated. Because this interaction was not retained in the final model, the resulting model represents differences in longevity between treatment groups while controlling for the additional effect of the number of clutches on longevity. Females that were not aged were not included in longevity models. Saturated models were reduced using the “step” function; model comparisons were conducted using χ^2^ analysis. Final LMs were validated by extracting model residuals using the “resid” function and testing them for normality using the Shapiro–Wilk test.

## 3 RESULTS

### 3.1 Effects on fecundity

**Figure 1:**
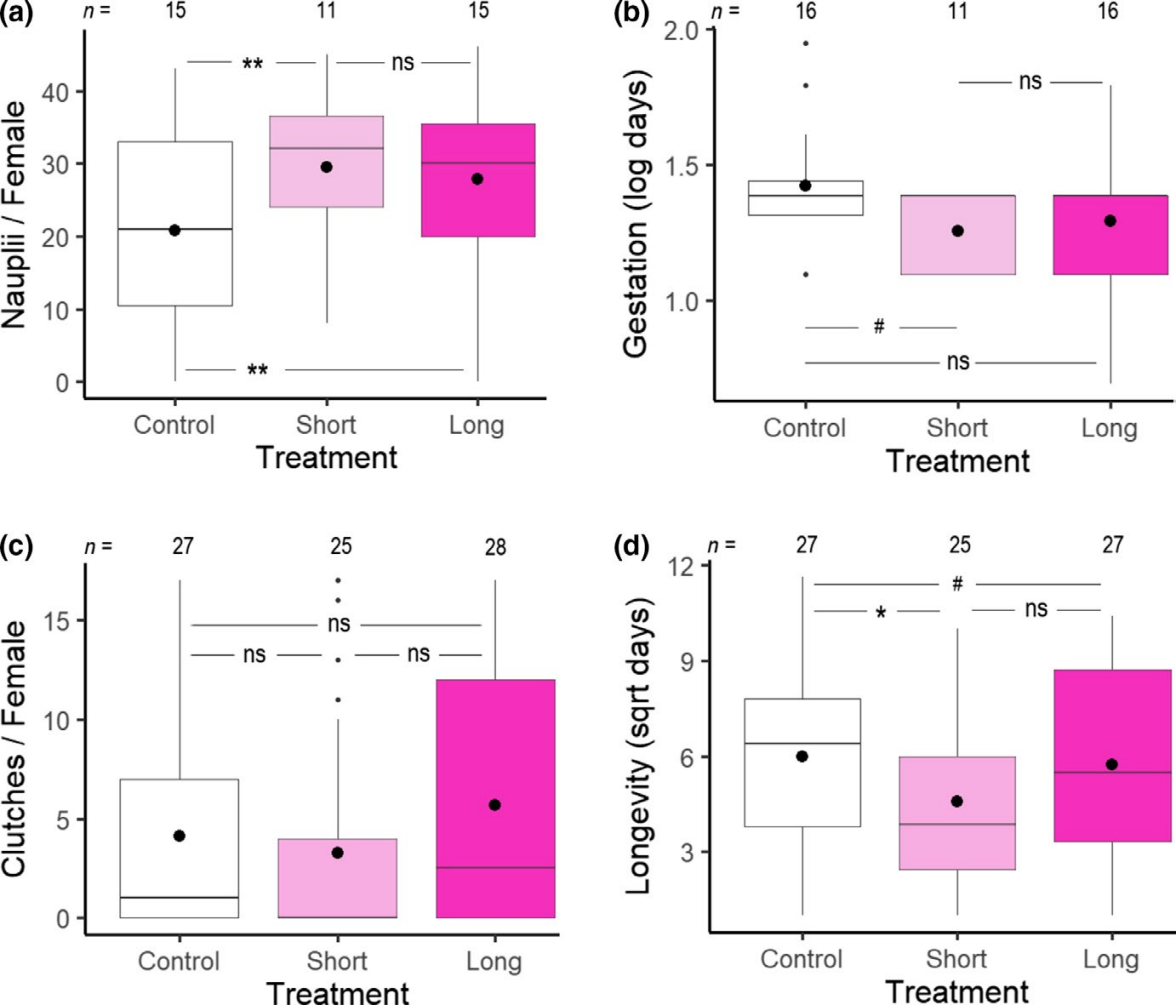
Boxplots showing effects of UV irradiation treatments on fecundity in female copepods, as indicated by (a) the number of nauplii produced in the first clutch with gestation duration as a covariate and (b) first‐clutch gestation duration. The effects of UV irradiation on reproductive effort are indicated by (c) the number of clutches (eggs sacs) produced per female, and additionally, the impact of treatment on (d) longevity with the number of clutches and age as covariates. Large dots represent mean estimates, and *n* is sample size. Significance codes: ns—not significant, ^#^0.1, *0.05, **0.01, and ***0.001

**Figure 2:**
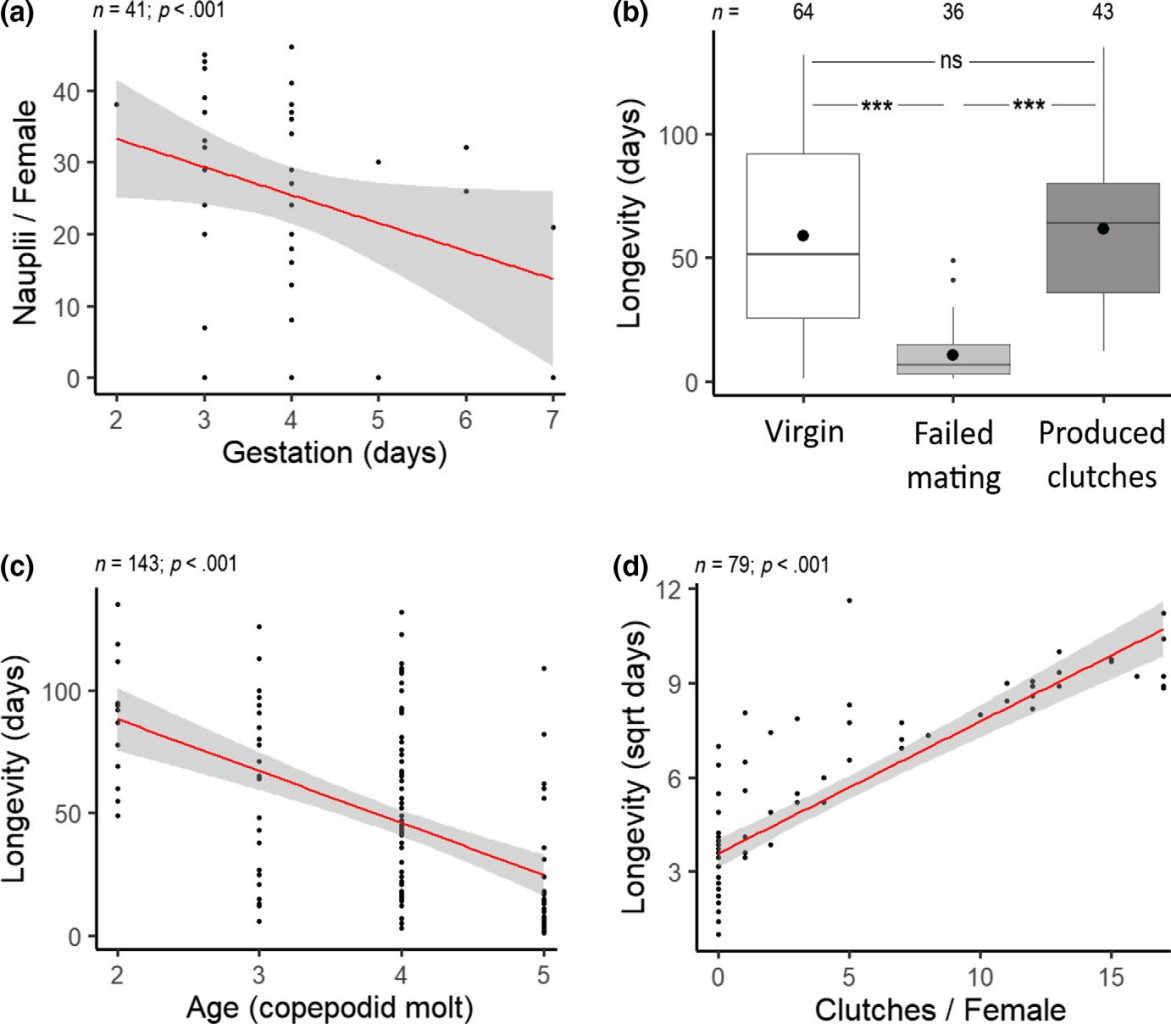
Scatterplots and boxplot indicating (a) the relationship between the number of nauplii produced in the first clutch and gestation duration with UV irradiation as a covariate, (b) the relationship between longevity and reproduction of virgin female copepods and females that mated and did or did not (failed mating) produce clutches with age as a covariate, (c) the relationship between longevity and age with reproduction as a covariate, and (d) the relationship between longevity and the number of clutches with age and UV irradiation as covariates. Large dots represent mean estimates, and *n* is sample size. Gray shading in scatterplots denotes 95% confidence intervals. Significance codes: ns—not significant, ^#^0.1, *0.05, **0.01, and ***0.001

Descriptive statistics are presented in Table 1. Of females that mated and produced a first clutch, the number of nauplii produced was significantly greater for 1‐ and 3‐hr UV treatments relative to the control while controlling for gestation duration (Table 2A; Figure 1a). Clutch size did not differ between 1‐ and 3‐hr UV exposure (Est. = −0.04; *SE* = 0.07; *p* = .58). Clutch size significantly decreased with increasing gestation duration (Table 2A; Figure 2a). Lastly, there was a trend suggesting that 1‐hr UV exposure could reduce gestation duration, but this was not statistically significant (Table 2B; Figure 1b). Gestation duration under 3‐hr UV exposure did not differ from the 1‐hr treatment (Est. = 0.03; *SE* = 0.09; *p* = .69).

**Table 1:** Means, standard deviations, and sample sizes (*n*) of life history responses in copepods with respect to UV irradiation treatment

Response	*n*	Control	1‐hr UV	3‐hr UV
Number of nauplii	41	20.8 ± 14.6	29.5 ± 10.9	27.8 ± 12.7
Gestation duration (days)	43	4.3 ± 1.3	3.5 ± 0.5	3.7 ± 0.9
Number of clutches	80	4.1 ± 5.3	3.2 ± 5.4	5.6 ± 6.6
Longevity (days)				
Virgins	64	61.3 ± 36.3	58.2 ± 40.1	57.1 ± 36.0
Failed mating	36	15.3 ± 16.2	8.5 ± 8.2	8.7 ± 5.9
Produced clutches	43	64.5 ± 34.1	54.4 ± 28.4	63.9 ± 27.5

Note

Under the longevity response, virgins represent females that did not mate, failed mating represents females that mated but did not produce clutches (egg sacs), and produced clutches represents females that mated and produced clutches.

**Table 2:** Results of GLMs predicting variation in the number of nauplii produced in the first clutch and the total number of clutches produced per female copepod

Response	Predictor	*n*	Est./*SE*
Number of nauplii^A^			
	1‐hr UV	11	0.24/0.08**
	3‐hr UV	15	0.20/0.07**
	Gestation duration	41	−0.13/0.03***
Gestation duration (days)^B^			
	1‐hr UV	11	−0.16/0.09^#^
	3‐hr UV	16	−0.13/0.08
Number of clutches^C^			
^1^ *Count model*	1‐hr UV	25	0.07/0.34
	3‐hr UV	28	0.39/0.31
^2^ *Zero‐inf model*	1‐hr UV	25	0.69/0.62
	3‐hr UV	28	0.18/0.60
Longevity (days)^D^			
	1‐hr UV	—	—
	3‐hr UV	—	—
	Virgin	64	37.75/6.51***
	Produced clutches	43	37.72/7.26***
	Age	143	−12.68/3.09***
Longevity (days)^E^			
	1‐hr UV	25	−0.93/0.40*
	3‐hr UV	27	−0.71/0.41^#^
	Number of clutches	79	0.34/0.03***
	Age	79	−0.80/0.21***

Note

Mean estimates of gestation duration (appearance of an egg sac to hatching) and longevity are presented from LMs. *n* is sample size, Est. is the point estimate, and *SE* is the standard error of the estimate. Mean estimates for 1‐ and 3‐hr UV irradiation treatments are estimated in comparison to controls. Longevity was modeled using two methods: one model with reproductive status (virgin, failed mating, and produced clutches) as a covariate and one model with the number of clutches that mating females produced as a covariate. Virgins and females that produced clutches are in comparison to females that mated but did not produce clutches (failed mating). Significance levels:

^#^ 0.1

* 0.05

** 0.01

*** 0.001

— not retained

### 3.2 Effects on reproductive effort and longevity

To determine the effects of UV irradiation on reproductive effort, we quantified the number of clutches produced by all females that mated. We also quantified the longevity of both mated and virgin females. Of females that mated, UV exposure had no effect on the number of clutches produced (Table 2‐C1; Figure 1c), nor on the odds of not producing clutches (Table 2‐C2).

To avoid collinearity, we modeled longevity using two different methods: first, with reproductive status (virgin, failed mating, or produced clutches) as an independent variable, and second, with the number of clutches produced by mated females as an independent variable. Females that mated but failed to produce clutches had significantly reduced longevity relative to both virgin females and females that did produce clutches (Table 2D; Figure 2b). The longevity of virgin females did not differ from the longevity of females that produced clutches (Est. = 0.03; *SE* = 5.70; *p* = .99). UV treatment was not retained in this model. While controlling for the number of clutches, longevity was lower following 1‐hr UV irradiation (Table 2E; Figure 1d). Longevity under 3‐hr UV exposure did not differ from the 1‐hr treatment (Est. = 0.22; *SE* = 0.41; *p* = .59). Age at the time of irradiation and longevity were inversely related in both longevity models (Table 2D,E; Figure 2c). The number of clutches produced increased significantly with longevity (Table 2E; Figure 2d).

## 4 DISCUSSION

An important focus of life history research is understanding how natural variation in organism performance arises within and between populations. The ability of organisms to survive to reproductive age and produce viable offspring is affected by numerous endogenous and exogenous factors (Devreker, Souissi, Winkler, Forget‐Leray, & Leboulenger, 2009; Fisher, 1930; Stearns, 2000). UV radiation is an exogenous, environmental factor that is known to play a significant role in the survival and reproduction of small‐bodied invertebrates such as copepods (Bidigare, 1989; Caramujo, Carvalho, Silva, & Carman, 2012; Damkaer, Dey, Heron, & Prentice, 1980). Accordingly, the aim of this study was to determine if UV irradiation can benefit organism performance in *T. californicus* copepods. We hypothesized that UV irradiation increases organism performance through enhanced fecundity, longevity, and reproductive effort. Our findings indicate that UV irradiation increased the number of nauplii produced from the first clutch of *T. californicus* females but also decreased the longevity among females that mated (Figure 1a,d). Our results may be explained by any single or combination of the following mechanisms: hormesis, antagonistic pleiotropy, or a reduction in pathogen load.

Under hormetic theory, low levels of exposure to a stressor improve organismal function, where higher levels of exposure decrease organism performance (Handy & Loscalzo, 2012; Hood et al., 2018; Mattson, 2008; Ristow, 2014; Yun & Finkel, 2014; Zhang et al., 2017). We speculate that the increase in copepod fecundity in both 1‐ and 3‐hr treatment groups suggests that the proximate, cellular benefits associated with modest UV irradiation (increased antioxidant production, signaled by increased oxidative stress) may have manifested in improved organism performance (Ristow, 2014; Zhang & Hood, 2016). The intensity of UV irradiation used in our study is similar to that of Han et al. (2016). In their study, Han et al. showed that other *Tigriopus* copepods exposed to 3 and 6 hr of UV irradiation at this intensity showed a rapid stress response at 96‐hr post‐treatment. Not only did ROS production increase significantly in ovigerous females in their study, but the production of antioxidant enzymes increased for both 3‐ and 6‐hr treatments, but not a 1‐hr treatment. Given that ROS was not upregulated at 1 hr in Han's study, but we found both an increase in nauplii production and decrease in longevity at this time point, other mechanisms could be at play. Souza, Hansson, Hylander, Modenutti, and Balseiro (2012) have also shown that freshwater copepods exposed to 38.9–233 kJ/m^2^ of UV‐A irradiation can elicit a short‐term stress response by upregulating the production of enzymes that counteract peroxidation, cell death, and enable neurotransmissions. While the 1‐hr UV treatment in our study was associated with increased fecundity, it was also associated with reduced survival. As such, the benefits of ROS exposure to fecundity may not have been enough to overturn oxidative damage that may be responsible for reduced longevity, although other mechanisms are possible. We also observed a decrease in longevity among females that mated but did not produce clutches. This may be due to poor condition of select females entering the study—irrespective of UV irradiation—ultimately leading to poor reproductive performance.

Alternate forms of radiation and stress have also been shown to elicit hormetic effects in other organisms (Shephard et al., 2018; Zhang et al., 2017). Shephard et al. (2018) recently characterized the effect of ɣ‐radiation on reproductive performance in the cricket *Acheta domesticus*. Similarly, they found that modest irradiation was associated with an increase in fecundity. While longevity was not reported, they also found an increase in average egg size. This result, along with the reduced gestation duration found in our study, suggests that radiation exposure may allow females to increase the quality of young, in addition to the quantity produced. In addition to exogenous, environmental stressors, endogenous stressors have also been found to benefit organismal performance. Work by Zhang et al. (2018) indicated that female mice that ran on a wheel before breeding produced more pups that were heavier at weaning than females that did not have a wheel. Consistent with hormetic theory (Hood et al., 2018; Mattson, 2008; Yun & Finkel, 2014), low exposure to a stressor in their study was shown to increase mitochondrial density in several organs and may be associated with increased organism performance. While modest levels of a stressor may be immediately beneficial, it is feasible that damage from oxidative stress may accumulate and have delayed impacts on performance and offspring quality (Rodríguez‐Graña, Calliari, Tiselius, Hansen, & Sköld, 2010).

Our study indicates that UV irradiation may increase reproduction early in life but also increase the rate of senescence later in life. In this respect, UV radiation may hold an adaptive significance and partly explain the short life cycles of copepods if individuals are able to increase their reproductive output at an early age (see Fernández, Campero, Uvo, & Hansson, 2018 for an example in cladocerans; Hylander et al., 2014). Trade‐offs between reproduction and longevity are predicted under antagonistic pleiotropy and the disposable soma theory of aging. Under antagonistic pleiotropy, selection is predicted to favor genes responsible for shorter lifespan when they are linked to increased reproductive success early in life (He & Zhang, 2006; Williams, 1957). Therefore, it is feasible that a gene, or suite of genes, is responsible for improved fecundity under UV irradiation. The disposable soma theory of aging states that allocating more resources to reproduction can reduce the allocation of resources to processes that support self‐maintenance and longevity (Gavrilov & Gavrilova, 2002; Kirkwood, 1977; Nussey, Kruuk, Donald, Fowlie, & Clutton‐Brock, 2006). Thus, it is feasible that the hormetic response to UV irradiation pulls resources away from maintenance, reducing longevity. Each of these mechanisms is speculative and warrant further investigation.

Finally, there are several other mechanisms that may be responsible for the observed effects. Pathogen infection of zooplankton—including copepods—is a widespread phenomenon in both freshwater and marine environments (Overholt et al., 2012; Seki & Fulton, 1969). Although UV radiation is often deemed detrimental to the life history of organisms, its effects may benefit host survival and/or reproductive performance by decreasing the survivability and prevalence of pathogens (Williamson et al., 2017). Evidence for a reduction of pathogen prevalence following UV irradiation has been supported in a fungal parasite of water fleas (Overholt et al., 2012), the bacterial load of rotifers (Munro, Henderson, Barbour, & Birkbeck, 1999), a nematode parasite of moths (Gaugler & Boush, 1978), and reduction of human parasites by UV‐B irradiation in vitro (Connelly, Wolyniak, Williamson, & Jellison, 2007), among other examples. Pathogen infection of copepods can decrease fecundity, egg production, respiration rates, and increase mortality (Albaina & Irigoien, 2006; Fields et al., 2014; Kimmerer & McKinnon, 1990). Aside from reducing the prevalence of pathogens, UV radiation can also influence reproduction in accordance with diet. Work by Hylander et al. (2014) has shown that female reproductive performance can increase following sublethal exposure to UV irradiation when females are fed a diet rich in mycosporine‐like amino acids that aid in screening UV radiation. Although diet may have had an influence on the ability of female copepods to defend against harmful UV irradiation in our study, it is unlikely that the observed effects herein are due to diet, provided that all females were supplied ad libitum access to the same algae throughout the study (see Lee et al., 2018 for how caloric restriction may influence the life history of aquatic organisms). Furthermore, UV radiation is necessary for endogenous vitamin D production; an induced increase in vitamin D via UV irradiation in *Daphnia* has been shown to increase fecundity (Connelly et al., 2015). However, previous work has demonstrated that vitamin D is likely not present in copepods (Karlsen et al., 2015).

We show that UV irradiation had an immediate, positive impact on fecundity, increasing the number of nauplii that females produced in their first clutch. This finding indicates that females exposed to UV radiation prior to reproducing may have an increased capacity to produce more offspring, at least early in their reproductive lifetime. Additionally, females with larger clutches also displayed relatively shorter gestation periods than females with smaller clutches. Reduced gestation could be associated with more rapid development or constraints on egg sac capacity. Further work is needed to determine if the total number of nauplii produced over a female's lifetime also increases significantly under UV irradiation. Additionally, our study has tested the effects of UV irradiation in a marine species of copepod (*T. californicus*) that exists above the intertidal zone along the west coast of North America. These copepods exist in shallow splash pools and are likely exposed to greater amounts of UV radiation in natural environments than in this study or other species of lake and ocean‐dwelling taxa of zooplankton. Alonso, Rocco, Barriga, Battini, and Zagarese (2004) and Overholt, Rose, Williamson, Fischer, and Cabrol (2016) demonstrate how copepods may exhibit UV radiation avoidance behavior, which may ultimately expose individuals to low‐intensity, UV radiation. As indicated by Williamson et al. (2019), the effects of ROS may only be relevant in species that exist within the top few centimeters of aquatic environments. Therefore, further research is needed to determine the likelihood that cellular signaling or ROS plays any role in the responses observed herein. If possible, future studies may also benefit from using natural sunlight to determine the effects of UV radiation on reproductive performance and life history characteristics (Williamson et al., 2019). Provided our aim was to demonstrate that UV irradiation can benefit organism performance, future work should aim to identify the proximate mechanisms that underly both the organismal benefit (increased fecundity) and detriment (decreased longevity) of UV irradiation.

## CONFLICT OF INTEREST

None declared.

## AUTHOR CONTRIBUTIONS

KBH, MJP, CK, and WRH conceived the study; KBH, CK, and VLT collected the data; KBH analyzed the data; KBH and WRH drafted the paper; all authors contributed to reviewing the final draft of the manuscript and agree to be accountable for all work published herein.

## Data Availability

Data are available from Dryad Digital Repository at https://doi.org/10.5061/dryad.kg18b9c.
